# Editorial: The progress of rare lesions of the sellar region

**DOI:** 10.3389/fendo.2022.978284

**Published:** 2022-08-24

**Authors:** Congxin Dai, Run Yu, Haijun Wang, Justo P. Castaño

**Affiliations:** ^1^ Department of Neurosurgery, Beijing Tongren Hospital, Capital Medical University, Beijing, China; ^2^ David Geffen School of Medicine, University of California, Los Angeles, CA, United States; ^3^ Department of Neurosurgery, The First Affiliated Hospital of Sun Yat-sen University, Guangzhou, China; ^4^ Maimonides Biomedical Research Institute of Córdoba (IMIBIC), Reina Sofia University Hospital, CIBERobn, University of Córdoba, Córdoba, Spain

**Keywords:** rare lesions of the sellar region, diagnostic molecular markers, pathogenesis, surgery, targeted therapies, radiotherapy, chemotherapy

Lesions of the sellar region are a heterogeneous group of diseases with complex clinical manifestations. The common lesions of the sellar region include pituitary adenomas (PAs), craniopharyngiomas (CPs), Rathke’s Cleft Cysts (RCCs), germ cell tumors, etc. (1–3). Rare lesions of the sellar region include PAs associated with McCune-Albright syndrome (MAS), or multiple endocrine neoplasia type 1 (MEN1), pituitary carcinomas (PCs), clival chordoma, pituitary metastasis from other tumors, giant invasive PAs, giant CPs, etc. (4, 5). These rare lesions of the sellar region present with complex clinical and atypical imaging features. Precisely diagnosing these rare lesions of the sellar region before surgery is notoriously difficult (6, 7). Accordingly, the challenge of diagnosing and treating these rare lesions of the sellar region, prompted us to devise a Research Topic related to the current progress on rare lesions of the sellar region.

In the present Research Topic in Frontiers in Endocrinology, we collected 22 studies and case reports on rare lesions of the sellar region. This Research Topic consists of two parts, the first part included nine articles of case reports that described various rare lesions of the sellar region. (i) IgG4-related inflammatory pseudotumors are one of the rare lesions of the sellar region, which are often misdiagnosed as other diseases. Liu et al. reported a case of an IgG4-related inflammatory pseudotumor involving the clivus mimicking meningioma. A male presented with intermittent headache for 2 years, and sudden dysphagia and hoarseness for 1 week. The magnetic resonance imaging (MRI) revealed an enhanced lesion located at the middle of the upper clivus region, which was preoperatively diagnosed with meningioma. The lesion was partially resected by endoscopic transsphenoidal surgery (TSS), and was pathologically diagnosed as IgG4-related inflammatory pseudotumor. Glucocorticoid was used postoperatively, and the patient’s symptoms including dysphagia and dysphonia were improved. Therefore, although the IgG4-related inflammatory pseudotumor is a rare lesion of the sellar region, it should also be considered a preoperative differential diagnosis. (ii) Primary pituitary lymphoma (PPL) is an extremely rare lesion of the sellar region with poor prognosis. Zhang et al. presented two cases of PPL and guided treatment and predicted prognosis using genetic analysis. The TP53 mutation and BCL6-LPP fusion were identified in one case of PPL by genetic analysis of cerebrospinal fluid and tumor tissue, and may be used as a marker for prognosis of PPL. Genetic analysis thus should be considered as a novel approach for prognosis prediction and treatment for PPL. (iii) Pituitary metastasis is an unusual and very rare disease, which accounts for approximately 1% of all intracranial metastases. It is difficult to diagnose the asymptomatic pituitary metastasis especially for patients with unknown primary malignant origin. Liu et al. reported a case of a male patient with visual changes and diabetes insipidus, and PA was initially diagnosed according to the MRI results with an extensive mass in the sellar region. The lesion of sellar region was completely resected though TSS, and the pathological results suggested the resected tumor tissue was pituitary metastasis from lung neuroendocrine tumor. Chest CT scan confirmed a pulmonary mass consistent with the primary neoplasm. Preoperative differentiation of pituitary metastases from PAs is very challenging because their clinical and radiological features are very similar. This case highlights the importance of a preoperative differential diagnosis of invasive lesions of the sellar region. (iv) Mature Cystic Teratoma (MCT) is a subset of intracranial germ cell tumors, and rarely occurs in the suprasellar region. Jiang et al. presented a unique and rare case of suprasellar MCT mimicking RCC. A young female patient complained of intermittent headache and oligomenorrhea for 11 years, and MRI demonstrated an irregular suprasellar lesion with slight intrasellar extension without obvious gadolinium enhancement. Endoscopic TSS was performed to remove the suprasellar lesion. According to the clinical, radiological, and operative findings, RCC was initially considered. However, the postoperative pathological findings revealed a mature cystic teratoma. Because the suprasellar MCTs are extremely rare, and their clinical and imaging features usually lacks specificity, it difficult to distinguish MCT from other cystic lesions located in the suprasellar region, and the precise diagnosis of MCT often relies on histological diagnosis. This case highlights the importance of obtaining a histological diagnosis to differentiate MCT from other lesions. (v) Rhabdomyosarcoma (RMS) is a malignant skeletal muscle sarcoma that rarely occurs intracranially, especially within the sellar region. Lu and Chen reported a pituitary RMS arising from a pituitary macroadenoma and its molecular profiling. They described an elderly man with an incidental discovery of a pituitary macroadenoma by MRI and underwent radiotherapy. Three years after radiation treatment, the tumor size increased significantly and caused mass effect on the optic chiasm. Transnasal endoscopic resection of the pituitary mass was performed, and a pituitary adenoma along with a separate spindle-cell sarcomatous component was identified by histological studies. Molecular profiling of the tumor using NextGen Sequencing identified mutations in TP53, ATRX, LZTR1, and NF1. This is the first case to report molecular features of a pituitary RMS arising from PA, which will help better understand its pathogenesis and to provide precise treatment plans. (vi) Extraventricular neurocytoma (EVN) located in the sellar/suprasellar region is an extremely rare tumor, and its biological behavior is unclear. Zhang et al. retrospectively analyzed the clinicopathological and molecular features of 4 cases of EVN arising from the sellar/suprasellar region, and demonstrated that EVNs arising from the sellar/suprasellar region had similar morphological features and immunophenotypes to classic central neurocytoma. However, there was no amplification of MYCN or EGFR, or no alterations in IDH1, IDH2, BRAF V600E, 1p/19q, H3F3A or CDKN2A were found in these tumors by molecular genetic testing. (vii) Granular cell tumors (GCT) of the pituitary are extremely rare lesion of the sellar region that arise from the posterior pituitary gland. Hong et al. reported a case of GCT of the pituitary and its genomic analysis results. An elderly female underwent resection of an incidentally diagnosed pituitary mass causing radiographic compression of the optic nerves. Histopathological findings of the resected tumor demonstrated a granular cell tumor of the posterior pituitary gland. The genomic analysis revealed mutations in key oncogenes such as SETD2 and PAX8, which have been reported in other cancers. This is the first report on comprehensive genomic characterization of granular cell tumor of the posterior pituitary gland. These data revealed feasible molecular pathogenesis of GCT and provided potential targeted therapies. (viii) Sellar glomus tumors are extremely rare lesions, which may be easily misdiagnosed as PAs. Cheng et al. reported a case of sellar glomus tumor which was misdiagnosed as NFPA. An elderly man presented with sellar mass for over 5 years and visual deficits for about 3 months. MRI revealed a lesion with cystic structures in the sellar region that was heterogeneously enhancing, and macroadenoma was initially diagnosed.  The sellar mass was resected via the TSS, and histopathological examination indicated a glomus tumor. This case suggests that glomus tumor should be considered as a differential diagnosis for sellar mass, and digital subtraction angiography examination is recommended before surgery. (ix) Although surgery is the first choice of treatment of CPs, it is associated with high long-term morbidity especially for CPs with hypothalamic involvement. Calvanese et al. reported that the BRAF/MEK inhibitor remarkably reduced tumor volume in two cases of papillary CP with confirmation of BRAFV600E mutation by biopsy. Therefore, BRAF/MEK inhibitor therapy may be a very useful and promising alternative treatment for giant and invasive CPs harboring the BRAFV600E mutation.

The second part included thirteen clinical studies that addressed the clinical characteristics and treatment outcome of lesions of the sellar region. (i) MAS is a disorder characterized by fibrous dysplasia, hyperfunctioning endocrinopathies and café-au-lait-spot. The coexistence of precocious puberty (PP) and growth hormone (GH) excess in patients with MAS is extremely rare. Zhai et al. reviewed seven MAS patients with GH excess and PP and found that all patients have craniofacial fibrous dysplasia-induced optic and otologic complications, increased growth velocity and advanced skeletal age. Early control of GH excess and PP leads to a reduced growth velocity and stabilized bone age. (ii) Cushing’s disease (CD) and ectopic ACTH syndrome (EAS) are two main causes of ACTH-dependent Cushing’s syndrome (CS). How to differentiate CD from EAS remain challenging. Currently, the high-dose dexamethasone suppression test (HDDST) was used to differentiate CD and EAS, low-dose dexamethasone suppression test (LDDST) was used to determine the diagnosis of CS. To develop an optimized pathway for the differential diagnosis of CD and EAS based on LDDST, Chen et al. performed a retrospective study of 261 CD and 29 EAS patients who underwent consecutive low- and high-dose DST. They found that LDDST with specific cut-off and HDDST had similar value to in differentiating CD from EAS, and combined LDDST and BIPSS could accurately differentiate CD from EAS. (iii) Basal ganglia germ cell tumors (BGGCTs) with or without sellar involvement is a group of very rare diseases. Zhang et al. evaluated the independent prognostic risk factors of patients with BGGCTs using multivariate analysis, and demonstrated that delayed diagnosis, focal radiotherapy, and non-pure germinoma were the independent poor prognostic risk factors of patients with BGGCTs. (iv) Adolescent thyrotropin-secreting adenoma (TSH-oma) in is a very rare kind of functional PA. Yang et al. summarized the clinical and therapeutic characteristics of 20 adolescent patients with TSH-oma, and found that these patients had larger tumors, higher TSH and thyroid hormone levels, and worse treatment outcomes than adult patients. (v) Large or giant PAs are relative rare tumors of the sellar region and remain therapeutically challenging.  Chen et al. explored the risk factors of recurrence or progression and predictors of prognosis of giant and large PAs after TSS, and indicated that partial resection, smoking, BMI **≧**25 kg/m^2^, Ki-67 ≥3%, and Knosp classification grade 4 increase the risk of the recurrence or progression of large and giant PAs. (vi) RCCs is benign and cystic disease of the sellar region, which often cause pituitary dysfunction. The relationship between clinical characteristics of RCCs and pituitary gland function is not very clear. Zhang et al. retrospectively analyzed 221 patients with RCC, and found that although pituitary function was not related closely to size of RCC, endocrine functions need to be evaluated for patient with RCC. (vii) Clival chordoma is a locally aggressive tumor of the sellar region. Chen et al. retrospectively analyzed the clinicopathological features and surgical outcomes of 17 consecutive patients with clival chordoma, and showed that endoscopic transsphenoidal surgery (ETS) is a safe and reliable approach for clival chordoma with high resection rates and low morbidity rates. (viii) Intracranial aneurysms (IAs) located at sellar region account for 10% to 20% among all the IAs and the co-existence of IAs and sellar region lesions is very rare and easily overlooked. Yan et al. retrospectively reviewed 515 continuous patients diagnosed with sellar region lesions and found the overall prevalence of sellar region lesions co-existed with the IAs is 11.1%. The patients with sellar region lesions and IAs exhibit higher comorbidity rate of endoscopic endonasal approach (EEA). Moreover, they suggested computed tomography angiography (CTA) as a routine preoperative examination for patients with sellar region lesions before EEA to guarantee surgical safety. (ix) Repeat surgery for recurrent or residual PAs (rrPAs) is technically challenging. To evaluate the safety and outcomes of ETS in rrPAs, Gong et al. reviewed clinical and radiological characteristics of 73 patients with rrPAs and investigated the factors influencing gross total resection (GTR) and intraoperative cerebrospinal fluid (CSF) leakage using logistic regression analyses. This study demonstrated that Knosp grade is an independent predictor of GTR in repeat ETS, and previous transcranial surgery and non-functional PAs are significantly associated with intraoperative CSF leakage. (x) Gangliocytomas/mixed gangliocytoma-adenomas (GCs/MGAs) are extremely rare lesions in the sellar region. Zheng et al. retrospectively studied the histological features of 4 patients with mixed gangliocytoma PAs and found that MGAs are associated with endocrinopathies such as acromegaly. Furthermore, they indicated that PIT1-positive PAs may have neural differentiation potential, which suggests that the neuronal transdifferentiation of adenomatous cells maybe a possible mechanism for MGA pathogenesis. (xi) It is difficult to resect completely PAs in Knosp grade 4, which are generally associated with poor prognosis. Guan et al. retrospectively reviewed the surgical outcome of transcranial approaches in the large-to-giant pPAs in Knosp grade 4 and found that 26.2% of them achieve gross total resection. Moreover, frontotemporal approach is more appropriate for tumors with large size and extending into the lateral skull base. In contrast, the frontotemporal approach was associated with longer surgical time and more bleeding volume. (xii) Chen et al. also retrospectively analyzed surgical outcomes of TSS in 239 patients with giant PAs in a single-center cohort and indicated that maximum diameter and Knosp grade of giant PAs have a significant impact on the extent of tumor resection. In addition, they found that the incidence of CSF leaks in neuroendoscopy group is significantly higher than that in the microscopic group. (xiii) Postoperative central nervous system infection (PCNSI) is an uncommon but serious complication of patient with sellar region tumors who underwent surgical interventions. Wen et al. identified risk factors for PCNSI via univariate and multivariate analyses, and indicated that transcranial surgery, intraoperative CSF leakage, postoperative diabetes insipidus and adrenal insufficiency are risk factors for PCNSIs

In summary, lesions of the sellar region are very complex, and a subset of them lack specific clinical and imaging features. Therefore, it is challenging to accurately diagnose these diseases before surgery, especially int the case of rare lesions. This Research Topic included a group of rare lesions of the sellar region that are easily misdiagnosed as other diseases. In addition, studies of surgical outcomes, risk factor analysis and molecular genetic testing for some sellar lesions were also included. Moreover, new therapeutic concepts and strategies such as personalized and targeted therapy also were reported. Through this Research Topic, we aimed at attaining a better understanding of rare lesions of the sellar region, and to provide new tools to diagnose and treat these diseases more accurately. We expect future issues will collect more studies on basic and translational research on lesions of the sellar region, bringing a better understanding of their characteristics and pathogenetic mechanisms to improve the clinical outcomes for these patients.

## Author contributions

CD and RY draft this manuscript. HW and JPC polished and revised the text. All authors contributed to the article and approved the submitted version.

## Funding

Financial support for this study was provided by the Beijing Municipal Administration of Hospitals Incubating Program (grant number: PX2022004). The funding institutions had no role in the design of the study, data collection and analysis, decision to publish, or preparation of the manuscript.

## Conflict of interest

The authors declare that the research was conducted in the absence of any commercial or financial relationships that could be construed as a potential conflict of interest.

## Publisher’s note

All claims expressed in this article are solely those of the authors and do not necessarily represent those of their affiliated organizations, or those of the publisher, the editors and the reviewers. Any product that may be evaluated in this article, or claim that may be made by its manufacturer, is not guaranteed or endorsed by the publisher.
